# Plastid Transformation of Micro-Tom Tomato with a Hemipteran Double-Stranded RNA Results in RNA Interference in Multiple Insect Species

**DOI:** 10.3390/ijms23073918

**Published:** 2022-04-01

**Authors:** Emine Kaplanoglu, Igor Kolotilin, Rima Menassa, Cam Donly

**Affiliations:** 1 London Research and Development Centre, Agriculture and Agri-Food Canada, London, ON N5V 4T3, Canada; emine.kaplanoglu@agr.gc.ca (E.K.); rima.menassa@agr.gc.ca (R.M.); 2 Solar Grants Biotechnology Inc., London, ON N6A 5R9, Canada; igor.k@sgbiotec.com; 3 Department of Biology, University of Western Ontario, London, ON N6A 3K7, Canada

**Keywords:** chloroplast dsRNA, cross-species RNAi, v-ATPaseA, droplet digital PCR, leaf-chewing insects, lacerate-and-flush feeding insects, sap-sucking insects

## Abstract

Plant-mediated RNA interference (RNAi) holds great promise for insect pest control, as plants can be transformed to produce double-stranded RNA (dsRNA) to selectively down-regulate insect genes essential for survival. For optimum potency, dsRNA can be produced in plant plastids, enabling the accumulation of unprocessed dsRNAs. However, the relative effectiveness of this strategy in inducing an RNAi response in insects using different feeding mechanisms is understudied. To investigate this, we first tested an in vitro-synthesized 189 bp dsRNA matching a highly conserved region of the *v*-*ATPaseA* gene from cotton mealybug (*Phenacoccus solenopsis*) on three insect species from two different orders that use leaf-chewing, lacerate-and-flush, or sap-sucking mechanisms to feed, and showed that the dsRNA significantly down-regulated the target gene. We then developed transplastomic Micro-tom tomato plants to produce the dsRNA in plant plastids and showed that the dsRNA is produced in leaf, flower, green fruit, red fruit, and roots, with the highest dsRNA levels found in the leaf. The plastid-produced dsRNA induced a significant gene down-regulation in insects using leaf-chewing and lacerate-and-flush feeding mechanisms, while sap-sucking insects were unaffected. Our results suggest that plastid-produced dsRNA can be used to control leaf-chewing and lacerate-and-flush feeding insects, but may not be useful for sap-sucking insects.

## 1. Introduction

RNA interference (RNAi) is a gene regulation and defense mechanism used by most eukaryotic organisms, including plants and insects [1,2]. Recently, it has also become a focal point in crop protection research, since it can be used to selectively down-regulate insect genes essential for survival [3]. The effector molecule that activates the RNAi pathway is double-stranded RNA (dsRNA), which triggers the post-transcriptional degradation of endogenous mRNA molecules having similar nucleotide sequences to the dsRNA [4]. To exploit the RNAi pathway for insect control, dsRNA can be produced in vitro, in bacteria or in plants, and then introduced orally to the insect [5,6,7]. Once present in the gut lumen, the ingested dsRNA molecules are taken up by gut cells through endocytosis or dsRNA-specific channels [8,9,10] and processed into small interfering RNA (siRNA) molecules of 21–23 nucleotides long by the RNAi machinery. The RNAi machinery then facilitates complementary base pairing between siRNA and target mRNA molecules, resulting in the subsequent down-regulation of the gene. If the gene is essential for insect survival, this can lead to insect mortality.

In order to be effective for crop protection, a potent RNAi response in the insect needs to be triggered, and to achieve this, several factors, including the choice of target gene, as well as the concentration, length, and stability of dsRNA in the insect gut and hemolymph, need to be taken into consideration [3,11,12]. Usually, the down-regulation of genes encoding essential products, such as actin, vacuolar-type H+ ATPase subunit A (v-ATPaseA), and tubulin, result in high mortality or severe developmental and physiological defects in insects [13,14,15,16]. However, though targeting these essential genes can achieve high mortality, it can also result in unintended cross-species effects on non-target organisms, as the nucleotide sequences of such genes are usually highly conserved, and the RNAi machinery does not require perfect homology between siRNA and the target mRNA for gene down-regulation [17]. Such cross-species effects can cause concerns over harming beneficial insects such as pollinators or predators.

In addition to the choice of target gene, the size of the dsRNA also plays an important role in RNAi effectiveness. For instance, long dsRNA molecules result in more efficient gene down-regulation than shorter molecules [18,19,20]. The stability of dsRNA in the insect’s gut is also an important factor, because it influences the dsRNA concentrations available for uptake by gut cells, which in turn affects RNAi success. For example, insects in the order Lepidoptera are recalcitrant to RNAi, and high concentrations of ingested dsRNA are required for gene down-regulation in lepidopterans because of the high activity of dsRNA-degrading nucleases in their gut lumen and hemolymph [14,21,22,23]. Furthermore, dsRNA can also be trapped in endosomes after being taken up by insect gut cells, which contributes to the poor RNAi response in some lepidopteran species [24]. In contrast, members of the order Coleoptera, including Colorado potato beetle (CPB) (*Leptinotarsa decemlineata*), are more responsive to RNAi, and have become model organisms for evaluating gene function in insects using RNAi [13,15,25,26,27]. The high efficiency of RNAi in coleopteran insects has been partially attributed to the presence of a coleopteran-specific protein (StaufenC) [28], which aids in the conversion of dsRNA to small interfering RNA (siRNA), a step required for the down-regulation of endogenous genes in insects.

Recently, modifying plant genomes has become a common approach for implementing RNAi for insect control, and many studies have used *Agrobacterium*-mediated nuclear transformation to produce dsRNA in plants [12,29]. However, this approach can be limited in its effectiveness, as dsRNA transcribed in the nucleus first enters the plant’s own RNAi pathway and is processed into siRNA molecules in the cytosol [6,16]. This subsequently reduces RNAi efficiency in the target insect, as siRNA uptake by insect gut cells is less efficient than that of long dsRNA [30]. To demonstrate that dsRNA processing in plants has this effect, the *Arabidopsis thaliana* RNAi pathway was de-activated by generating mutations in dsRNA processing enzymes, which led to an increased accumulation of unprocessed dsRNA, resulting in more efficient gene down-regulation in cotton bollworm (*Helicoverpa armigera*) [6].

To avoid the processing of dsRNA in the plant, molecules can be produced in the prokaryotically derived plastids of plants, which lack intrinsic RNAi processing machinery, thus allowing the accumulation of intact dsRNA [12,31]. This strategy was shown to provide full protection to potato plants against CPB, as dsRNA molecules remained unprocessed in the plants [16], leading to a greater reduction in mRNA levels in insects feeding on transplastomic plants compared to insects feeding on nuclear transformed plants. Similarly, a more potent reduction in mRNA levels and larval growth was observed in cotton bollworm when the insects were fed chloroplast-transformed *Nicotiana benthamiana* plants compared to nuclear-transformed plants [32].

Although plastid transformation holds great promise for insect control, the length of the dsRNA product to be accumulated and the feeding behavior of the target insect must also be considered. Previously, we demonstrated that a 2222 bp dsRNA targeting tobacco hornworm (*Manduca sexta*) *v*-*ATPaseA* gene accumulated in significantly lower quantities than a 223 bp *GFP* dsRNA in tobacco, implying a size-dependent limit on the quantities of dsRNA that can be produced in plastids [18]. Similar results were also demonstrated in potato, in which shorter dsRNA targeting the CPB β-Actin gene accumulated at higher quantities than the longer dsRNA of the same gene [20]. This can then have a significant impact on RNAi success in insects, especially in members of the order Lepidoptera, which require high concentrations of ingested dsRNA for gene down-regulation [14]. In addition, the mechanism by which insects feed can affect the availability of dsRNA for ingestion. For instance, some of the most crop-devastating insects from the order Hemiptera, including whiteflies, aphids, and mealybugs, feed on plant sap as their main or sole food source [33,34]. Since plastid-produced dsRNA is contained within the organelles, it might not be present within the vascular system of the plant and therefore not be available for ingestion by these sap-sucking insects [35].

In this study, our main goal was to assess the effects of plastid-produced dsRNA on insects that use a range of feeding mechanisms. However, since plastid transformation is quite time consuming [36,37], it was only practical to develop one transplastomic plant line for testing multiple insect species. Therefore, we selected one dsRNA sequence that can down-regulate the target gene in all species. Consequently, this meant that there was only a moderate match in the dsRNA sequence with the same gene in each insect. As a result, only moderate levels of target gene down-regulation were expected, which would result in a greater sensitivity when detecting differences in RNAi efficiency due to the feeding strategy. For our cross-species effective dsRNA, we selected a 189 bp in vitro-synthesized dsRNA based on a highly conserved insect gene, *v*-*ATPaseA*, from cotton mealybug (*Phenacoccus solenopsis*). v-ATPaseA is one of the fourteen subunits of multi-subunit v-ATPase proteins that function as ATP-dependent proton pumps in eukaryotic cells to maintain pH homeostasis as well as other vital functions [38]. The A subunit is an essential part of the pump as it contains the catalytic ATP binding sites; the down-regulation of the expression of this subunit has been shown to be lethal in many insect species when strong gene down-regulation is accomplished [13,18,39,40].

We first demonstrated the ability of the cotton mealybug *v*-*ATPaseA* dsRNA to down-regulate the *v*-*ATPaseA* gene in three target insects from two different orders that use feeding mechanisms such as leaf-chewing, lacerate-and-flush, and sap-sucking mechanisms. We then expressed the dsRNA in the plastids of Micro-tom tomato to develop transplastomic Micro-tom (tpMicro-tom) plants. We chose Micro-tom for plastid transformation, as opposed to commonly used tobacco, since tomato plants serve as a food source for a wide range of insect pests [41]. Next, we used droplet digital PCR (ddPCR) to determine the absolute quantities of dsRNA in various plant tissues. Finally, we fed the same three insect species having different feeding mechanisms with wild-type (WT) or tpMicro-tom leaves to test the down-regulation effects of the plastid-produced dsRNA in each species.

## 2. Results

### 2.1. Cotton Mealybug-Specific dsRNA Down-Regulates v-ATPaseA Gene in Three Insect Species

To determine whether a 189 bp dsRNA from cotton mealybug can down-regulate the *v*-*ATPaseA* gene in CPB, brown marmorated stink bug (BMSB) (*Halyomorpha halys*), and Madeira mealybug (MMB) (*Phenacoccus madeirensis*), in vitro-synthesized dsRNA was used. A 223 bp dsRNA matching a portion of a green fluorescent protein gene (*GFP*) was also used as a dsRNA control. To expose CPB to the dsRNA, potato leaves were dipped in dsRNA solutions or water, and first instar CPB larvae were allowed to feed on the treated leaves ad libitum, while BMSB and MMB adults were exposed to dsRNA or water via injection. After three days, the mRNA levels of the *v*-*ATPaseA* gene were analyzed using RT-qPCR.

**Figure 1:**
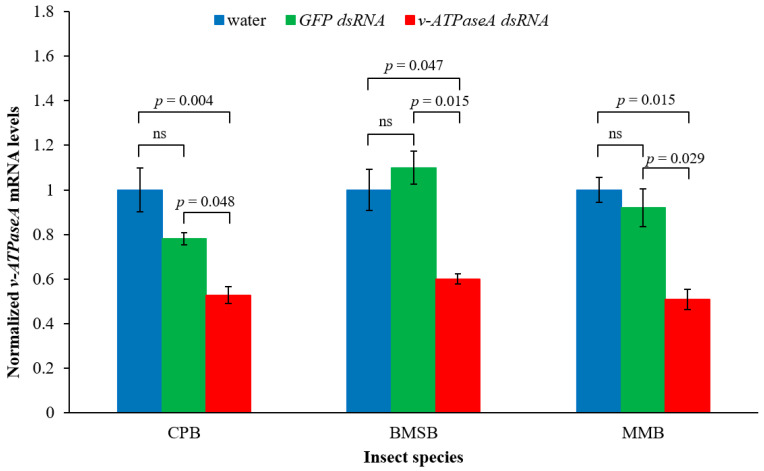
Down-regulation of *v*-*ATPaseA* gene in three insect species when treated with in vitro-synthesized 189 bp dsRNA based on cotton mealybug *v*-*ATPaseA*. CPB larvae were fed dsRNA or water coated on potato leaves while BMSB and MMB adults were injected with dsRNA or water. Normalized mRNA levels in water treatment were set to the value “1.0”, and the mRNA levels in *GFP* and *v*-*ATPaseA* treatments were calculated relative to the water treatment. RT-qPCR data are expressed as mean relative quantity ± SEM, *n* = 3. *p*-values were calculated using Tukey’s HSD (one-way ANOVA) for each comparison and are indicated on the graph. CPB = Colorado potato beetle; BMSB = brown marmorated stink bug; MMB = Madeira mealybug. “ns” = not significant (*p* > 0.05) according to Tukey’s HSD test.

In CPB, feeding on the 189 bp *v*-*ATPaseA* dsRNA resulted in 47.2% gene down-regulation compared to water-fed insects, which was significant according to ANOVA, followed by Tukey’s Honestly Significant Difference (HSD) post hoc analysis (*F* = 13.87, *p* = 0.0056, *n* = 3), while feeding on *GFP* dsRNA resulted in a small effect, which was not significant (*p* > 0.05) (Figure 1). Similarly, in BMSB and MMB adults, an injection of the 189 bp *v*-*ATPaseA* dsRNA resulted in 40.0% and 49.0% gene down-regulation, respectively, compared to water-injected insects, both of which are significant according to ANOVA, followed by Tukey’s HSD analysis (*F* = 9.14, *p* = 0.015, *n* = 3 for BMSB and *F* = 9.86, *p* = 0.013, *n* = 3 for MMB). The injection of *GFP* dsRNA did not result in gene down-regulation in either species (*p* > 0.05) (Figure 1).

### 2.2. Development of tpMicro-Tom to Express dsRNA

**Figure 2:**
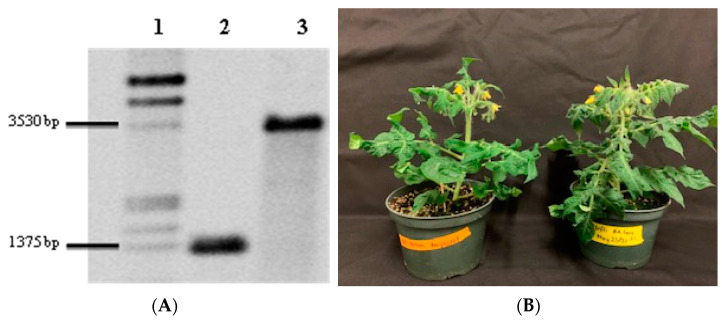
Southern blot analysis and phenotype of Micro-tom plants. (**A**) Southern blot confirming production of homoplastomic tpMicro-tom plants. Ten micrograms of total DNA was digested using *Mfe* I restriction enzyme, and the DNA fragments were separated on a 0.8% agarose gel for blotting. Lane 1 = Dig-labelled DNA ladder III (Roche); Lane 2 = WT Micro-tom (1298 bp); Lane 3 = tpMicro-tom (3353 bp). (**B**) Phenotype comparison of 8-week-old WT Micro-tom and T_1_ tpMicro-tom plants. The seeds were first germinated on Murashige and Skoog (MS) medium, and the plants were transplanted to soil after 3 weeks for further growth in a greenhouse.

Having demonstrated that in vitro-synthesized dsRNA specific for the cotton mealybug *v*-*ATPaseA* gene has significant cross-species effects on all three insect species used, we decided to produce the same dsRNA in Micro-tom plastids and to determine if plastid-produced dsRNA has the same effects when delivered in this way. To achieve this, plastid transformation was carried out using the biolistic particle delivery method, and the first transformed callus, which was white to pale yellow in color (Supplementary Figure S1A), was observed after 5 months. The callus turned green with repeated sub-culture on a regeneration medium (RM) to ensure homoplastomy (Supplementary Figure S1B). After being placed on a shoot-inducing medium (SIM), it took at least 2 months to observe shoots emerging from the callus (Supplementary Figure S1C). To ensure that tpMicro-tom was homoplastomic, a Southern blot analysis, using DNA extracted from the leaves of the primary transformants (T_0_), was performed. The results confirmed that the plants were homoplastomic, as all the restriction fragments were of expected sizes for the recombinant and the WT plastomes (WT Micro-tom = 1298 bp, and tpMicro-tom = 3353 bp; Figure 2A). Overall, it took about 17 months from the bombardment of leaves to obtain the first batch of T_1_ seeds from the tpMicro-tom expressing the desired dsRNA. The phenotype of tpMicro-tom plants was morphologically indistinguishable to WT Micro-tom (Figure 2B).

### 2.3. dsRNA Concentrations Are Higher in the Leaves Compared with Other Tissues

**Figure 3:**
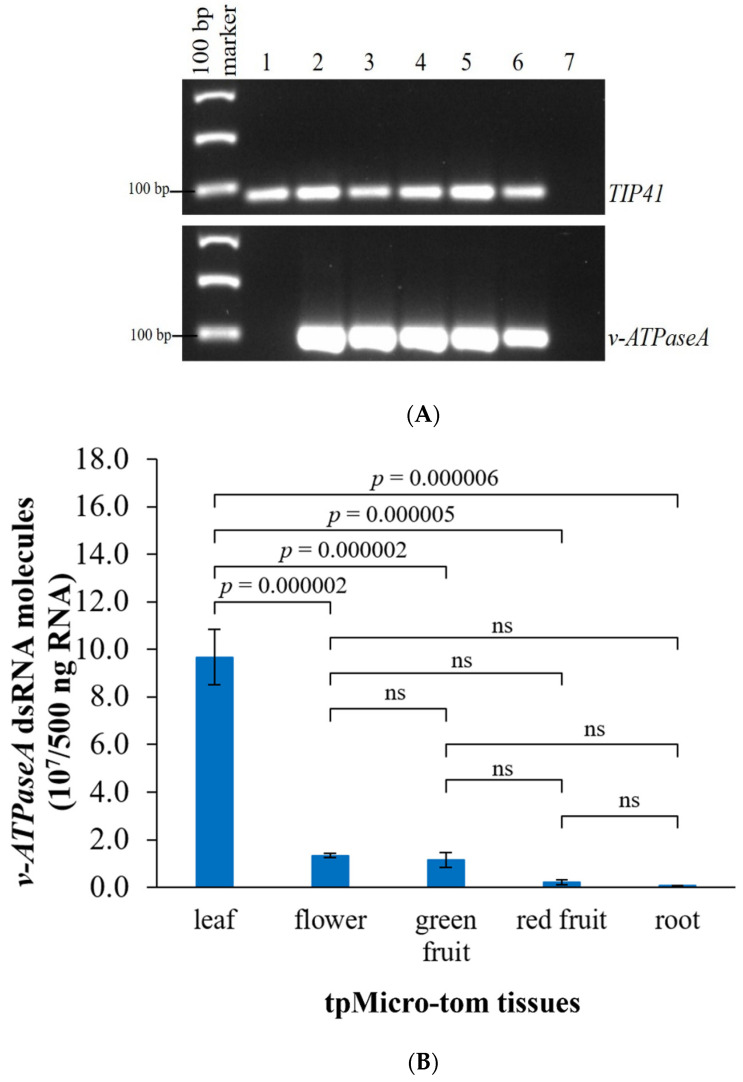
dsRNA production in different tpMicro-tom tissues. (**A**) Qualitative assessment of dsRNA production using end-point PCR and agarose gel that show dsRNA presence in various tpMicro-tom tissues; Lane 1 = WT leaf; Lane 2 = transplastomic (tp) leaf; Lane 3 = tp flower; Lane 4 = tp green fruit; Lane 5 = tp red fruit; Lane 6 = tp root; and Lane 7 = no template control. (**B**) Absolute quantification of *v*-*ATPaseA* dsRNA molecules in 500 ng tpMicro-tom total RNA using ddPCR. Data are expressed as mean quantity ± SEM, *n* = 3. *p*-values were calculated using Tukey’s HSD (one-way ANOVA) for each comparison and are indicated on the graph. “ns” = not significant (*p* > 0.05) according to the Tukey’s HSD test.

To confirm that the desired dsRNA is produced in various tissues (leaf, flower, green fruit, red fruit, and root) of tpMicro-tom plants, total RNA was extracted from WT Micro-tom and tpMicro-tom plants and reverse transcribed into complementary DNA (cDNA). Then, end-point PCR was performed using *v*-*ATPaseA* dsRNA-specific primers as well as primers specific for a Micro-tom reference gene, the TIP41-like family protein gene (*TIP41*) [42]. Using these primers, *TIP41* (control) was detected in both WT Micro-tom and tpMicro-tom cDNA, whereas *v*-*ATPaseA* dsRNA was only detected in tpMicro-tom cDNA (Figure 3A), confirming the production of dsRNA in the tpMicro-tom tissues. Next, ddPCR was performed using cDNA as a template to assess absolute quantities of dsRNA in different tpMicro-tom tissues. For each tissue, the samples were collected from three different tpMicro-tom plants to have three different biological replicates (*n* = 3). The dsRNA quantities were the highest in leaf, followed by flower, green fruit, red fruit, and root. Based on a rate of 100% conversion of dsRNA to cDNA, there were 9.67 × 10^7^, 1.33 × 10^7^, 1.14 × 10^7^, 2.06 × 10^6^, 6.20 × 10^5^ dsRNA molecules in 500 ng total RNA from leaf, flower, green fruit, red fruit, and root, respectively (Figure 3B and Table S3). The number of dsRNA molecules was significantly different in the tissues according to one-way ANOVA (*F* = 55.55, *p* < 0.001, *n* = 3), and a post hoc analysis using Tukey’s HSD test showed that tpMicro-tom leaves have significantly higher quantities of dsRNA compared to any other tissues tested (Figure 3B).

### 2.4. Insect Feeding Strategy Affects v-ATPaseA mRNA Levels Resulting from Exposure to Plastid-Produced dsRNA

**Figure 4:**
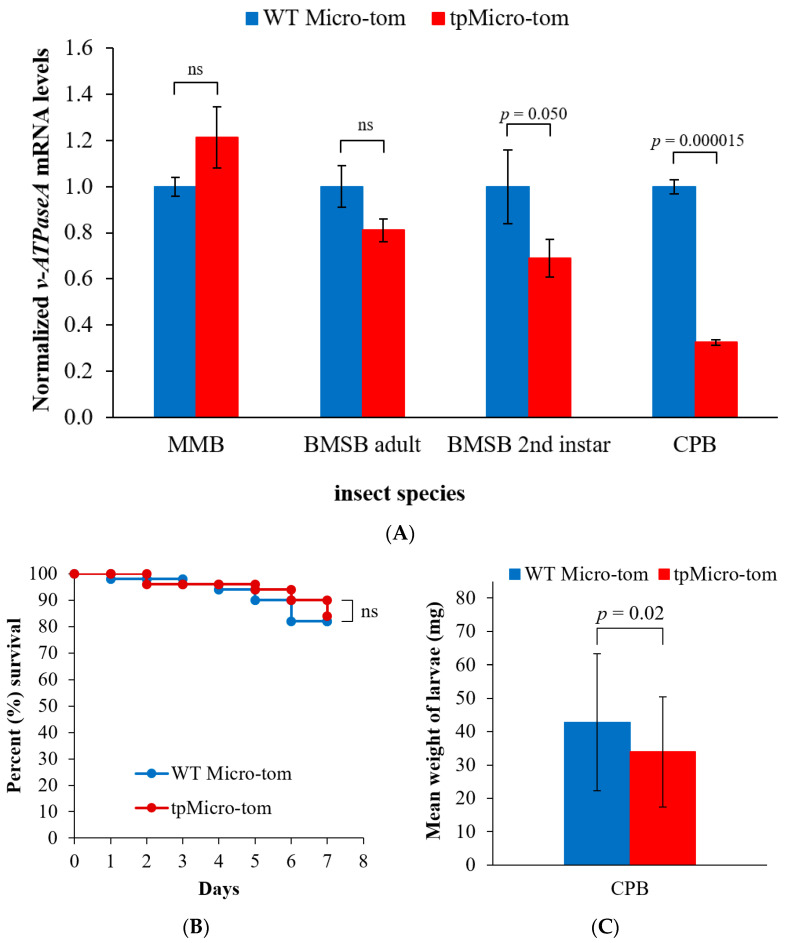
Normalized relative *v*-*ATPaseA* mRNA levels in different insect species after feeding on plastid-produced dsRNA, and survival and weight analysis for CPB. (**A**) mRNA levels for MMB adults, BMSB adults, BMSB 2nd instar larvae, and CPB 1st instar larvae. mRNA levels in WT Micro-tom-fed insects were set to the value “1.0”, and mRNA levels in tpMicro-tom-fed insects were calculated relative to the WT Micro-tom-fed insects. Reference genes used were *betaTUB* for MMB, *60S RP* for BMSB, and *RPL8E* for CPB. Data are expressed as mean relative quantity ± SEM, *n* = 3, and two-sample *t*-tests were used to determine *p*-values which are indicated on the graph. MMB = Madeira mealybug; BMSB = brown marmorated stink bug; CPB = Colorado potato beetle; “ns” = not significant (*p* > 0.05) according to *t*-tests. (**B**) Kaplan–Meier survival analysis for CPB larvae after 7 days of feeding on WT Micro-tom or tpMicro-tom. “ns” = not significant (*p* > 0.05) according to Log-rank post hoc analysis. (**C**) Mean weight of CPB larvae after feeding on WT Micro-tom or tpMicro-tom. Data are expressed as mean weight ± SD; *n* = 50. Two sample *t*-test was used to determine *p*-value which is shown on the graph.

To evaluate whether the insect feeding strategy would have an effect on the success of RNAi using dsRNA produced in plastids, three insect species were fed with WT Micro-tom or tpMicro-tom, and the *v*-*ATPaseA* mRNA levels were measured by RT-qPCR. For MMB adults, which feed by sap sucking, there was no difference in *v*-*ATPaseA* mRNA levels between the two diets (*p* > 0.05, *n* = 3) (Figure 4A) after 7 days of feeding. For BMSB, which use a lacerate-and-flush feeding strategy, there was a small reduction (19%) in *v*-*ATPaseA* mRNA levels in adults fed with plastid-produced dsRNA, but the reduction was not statistically significant (*p* > 0.05, *n* = 3) (Figure 4A). To investigate this further, we decided to repeat the experiments using second instar BMBS larvae, as RNAi can be more efficient in younger instars [43]. In this case, we observed a significant (31%) down-regulation of the *v*-*ATPaseA* gene (*t* = −2.8, *p* < 0.05, *n* = 3) in second instar BMSB larvae fed on the tpMicro-tom for 5 days compared to those fed on WT Micro-tom (Figure 4A).

Finally, for the leaf-chewing CPB, an mRNA levels analysis showed that *v*-*ATPaseA* gene was down-regulated by 67.5% in tpMicro-tom fed insects compared with WT Micro-tom fed insects, a significant change according to a two-sample *t*-test (*t* = −24.91, *p* < 0.001, *n* = 3) (Figure 4A). Additionally, since gene down-regulation was more effective in CPB, an insect survival bioassay and insect weight measurements were performed to assess whether such a down-regulation might result in mortality and/or weight reductions in tpMicro-tom-fed insects compared with WT Micro-tom-fed insects. No increase in mortality was observed in tpMicro-tom-fed CPB (*p* > 0.05, *n* = 50) (Figure 4B); however, a significant (*t* = 2.4, *p* = 0.02, *n* = 50) reduction was noted in the weight of tpMicro-tom-fed larvae compared with those fed WT Micro-tom leaves (Figure 4C).

## 3. Discussion

Producing dsRNA in the plastids of plants for insect pest control has great advantages, as the dsRNA molecules remain unprocessed and accumulate in high quantities, which can then increase RNAi efficiency in target insects. However, this strategy has been only lightly explored, and to date, only the plastids of potato and tobacco have been transformed for this purpose [12,18,32,35,44]. In addition, there is very limited knowledge on how this strategy can be affected by the feeding mechanisms of targeted insects. To address this knowledge gap, we need to identify a dsRNA sequence that could down-regulate a target gene in multiple insects that use different feeding mechanisms. Then, we needed to express the same dsRNA in the plastids of tomato plants to determine whether presenting the same dsRNA in plastids still resulted in gene down-regulation in the target insects.

To address these objectives, we first show that a dsRNA designed from the *v*-*ATPaseA* gene from cotton mealybug (order: Hemiptera) significantly down-regulates the *v*-*ATPaseA* gene not only in two hemipteran insects, namely BMSB and MMB, but also in CPB (Order: Coleoptera), when in vitro-synthesized dsRNA was provided via injections or feeding. Previously, several studies demonstrated the cross-species effects of dsRNAs targeting conserved genes on non-target insects [13,45,46,47,48,49]; however, only a few of these studies have investigated the potential RNAi effects on non-target insects belonging to different orders [45,50]. For instance, Bachman et al. (2013) investigated the insecticidal activity of a dsRNA targeting the coleopteran Western corn rootworm (*Diabrotica virgifera virgifera*) *Snf7* gene in various insects, belonging to 10 families in four different orders [45]. The study revealed that the dsRNA only had effects on coleopteran insects whose *Snf7* genes had sequence identities of more than 90% with that of the western corn rootworm, and suggested that the genes needed to share stretches of at least 21 identical nucleotides for RNAi to work in non-target insects. Contrary to this finding, Powell et al. (2017) showed that identical nucleotide sequence stretches of 15 bases were enough to induce cross-species effects of dsRNA in the order Diptera [48]. The dsRNA sequence we used in our study had sequence identities of 76% to 86% with the *v*-*ATPaseA* genes in insects used in this study (Table 1). Furthermore, identical nucleotide sequence stretches were 20, 20 and 14 bases with the *v*-*ATPaseA* genes of BMSB, MMB and CPB, respectively (Supplementary Figure S3A–C); hence, our results are in accordance with the findings of Powell et al. (2017) and provide further evidence of broader cross-species RNAi effects when conserved sequences are used in dsRNA design.

Having demonstrated that the 189 bp cotton mealybug-based dsRNA can successfully down-regulate the *v*-*ATPaseA* gene in three insect species, we then wanted to determine whether the same dsRNA can down-regulate the same gene when presented in plant plastids. For this purpose, we chose the tomato variety Micro-tom, as it can serve as a food source for all the insects we used in this study. Previously, most studies focused on using tobacco plants for plastid transformation, as it is more easily transformed due to availability of a well-established protocol [18,32,51]. However, because of the presence of nicotine, an alkaloid that can act as natural insecticide [52], tobacco is a host for fewer insect herbivores. Therefore, we chose tomato plants which are more facile for use in studying RNAi characteristics in a greater variety of insects. Compared with tobacco, transforming the plastome of tomato can be quite laborious, and leaf regeneration and transformation efficiency are highly dependent on the tomato genotype used [36,53,54]. So far, studies using Micro-tom tomato have mainly focused on *Agrobacterium*-mediated transformation using primarily cotyledons as starting materials [55,56]. Here, we used Micro-tom leaves for plastid transformation and showed that the process for plastome transformation of Micro-tom is attainable, albeit slow, as it took us about 17 months to obtain T_1_ seeds from the transplastomic lines. Previously, it was reported that the plastid transformation of another tomato variety (IAC-Santa Clara) took about two years [36], which was close to the timeframe we report for Micro-tom.

We also demonstrated, for the first time, that dsRNA molecules are produced in the leaf, flower, green fruit, red fruit, and root of the tpMicro-tom. Among the previous studies that used transplastomic plants for dsRNA production, only one study analyzed dsRNA production in tissues other than leaves, namely in the tubers of transplastomic potato [16]. The authors used northern blotting, but did not detect dsRNA in the tubers, probably due to the low sensitivity of the technique used. Here, using PCR, we demonstrated that dsRNA was present in all the plant tissues analyzed, consistent with the constitutive nature of the *rrn* promoter used in our transformation construct pTomCT [18,37]. Furthermore, using ddPCR, we determined the absolute quantities of dsRNA in tpMicro-tom tissues, and showed that the dsRNA concentration is significantly higher in the leaf compared with the other tissues tested. This result was expected as transcription rates in plastids are at their highest in photosynthetically active chloroplasts [57,58,59], which would result in a higher accumulation of dsRNA molecules in the leaves, compared with other tissues. Interestingly, a significantly lower level of dsRNA was detected in green fruit (~8.5 × reduction) compared with leaf tissue, despite the fact that green fruits also contain chloroplasts. This is most likely due to the fact that transcription in plastids is reduced significantly in green fruits compared with leaves, as a result of developmental programming [57]. Furthermore, we observed no significant difference in dsRNA quantities in red fruit tissue compared with green fruit tissue, which is in accordance with the limited changes in transcriptional activity in tomato fruit during ripening and chloroplast-to-chromoplast differentiation, as reported previously [57]. We also detected dsRNA in flower and root, although the levels were the lowest in the root among all the tissues tested. Hence, dsRNA quantities accumulating in root tissue plastids are probably not high enough to confer protection from pest insects that feed on the roots of plants.

In addition to tissue type, the concentrations of dsRNA produced in plastids also depend on dsRNA size, as smaller dsRNA molecules are shown to accumulate in much higher quantities than longer dsRNA molecules in plant plastids [18,20]. This is mainly believed to be due to the instability of long RNA molecules in plastids, since they tend to form secondary structures under cellular conditions [60], possibly preventing proper dsRNA formation after transcription. This, in turn, can make the long RNA molecules more susceptible to mechanical damage and degradation by nucleases [61]. In fact, we previously showed that a 223 bp dsRNA accumulated two times more than a 2222 bp dsRNA in tobacco plastids [18]. In addition, a study by He et al. (2020) compared the productions of 200, 297, 400 and 700 bp dsRNA in potato plastids using northern blotting and detected increased RNA degradation with increasing RNA sizes [20]. Based on their results, the authors concluded that using shorter dsRNA might achieve a better yield in dsRNA production in plastids, which in turn can provide better protection against insect pests. In our study, we used 189 bp dsRNA, which should accumulate in sufficient quantities in Micro-tom plastids to provide enough gene down-regulation to assess the effects of plastid-produced dsRNA on insects using different feeding mechanisms.

Previously, plastid-produced dsRNA was mainly used to down-regulate genes in insects that use leaf chewing as their feeding mechanism [16,18,44,62], as these insects naturally ingest the dsRNA-containing plastids during feeding. In this study, we used not only leaf-chewing insects, but also insects that use lacerate-and-flush and sap-sucking mechanisms to test the effects of plastid-produced dsRNA in insects using feeding mechanisms other than leaf chewing. With respect to leaf-chewing insects, we used CPB, and observed a significant down-regulation of the *v*-*ATPaseA* gene (67.5% reduction in mRNA levels). Although the down-regulation of the gene did not affect survival, significant weight loss in larvae was observed. The down-regulation of *v*-*ATPaseA* has been shown to have various effects, including mortality, weight reduction, and stunted growth in multiple insect species [13,18,27,44,63]; hence, it is likely that the degree of gene down-regulation was not enough to induce mortality in the larvae by the end of our 7-day bioassays. In fact, it was previously demonstrated that in CPB, the down-regulation of *v-ATPase* genes can result in mortality or weight loss depending on the degree of gene down-regulation [27].

With respect to lacerate-and-flush insects, we used BMSB, which uses its watery saliva to liquefy plants’ cellular contents and ingest the resulting juice produced [64]; hence, it is expected to consume the dsRNA produced in plant plastids. Our results showed that although BMSB adults were not significantly affected, plastid-produced *v*-*ATPaseA* dsRNA did have a significant effect on second instar BMSB larvae, with a 31% reduction in mRNA levels. This shows that BMSB is indeed able to ingest the dsRNA from plastids; however, some of the dsRNA is probably degraded by saliva during feeding, reducing the RNAi efficiency. In fact, a previous study demonstrated the presence of dsRNA-degrading nucleases in BMSB adult saliva and showed that the injection of dsRNA works better than ingestion for inducing gene down-regulation [65]. However, given that gene down-regulation was significant in second instar larvae, this may also indicate the life-stage dependency of RNAi in BMSB, especially when dsRNA is delivered in plant plastids. This phenomenon was also observed in other insects in which RNAi was more effective in young larvae compared to older larvae [43].

With respect to sap-sucking insects, we used MMB, which inserts its mouthparts into the vascular tissue of plants and ingests plant sap [33,34]. During feeding, sap-sucking insects, such as MMB, use their stylets (thin and elongated mouthparts) to weave their way around and between plant cells until the stylet reaches the sieve elements that contain sap [66]. By doing so, they avoid puncturing the cells on the plant surface and cause minimal damage to the cells that have dsRNA-expressing plastids (mainly chloroplasts). Hence, dsRNA remains in the plastids and does not leak into the cytoplasm of cells (from where the dsRNA could be potentially transported to sieve elements). Furthermore, although sieve elements also contain plastids, mostly P-plastids and S-plastids, these plastids are mainly used by plants to store protein and starch, respectively [67]. Therefore, sieve-element plastids are not expected to have active transcriptional machinery to produce dsRNA [68]. As a result, when dsRNA is produced in plant plastids, it will not be present in plant sap, and sap-sucking insects are not expected to ingest the dsRNA. As predicted, no down-regulation of the target gene was observed in MMB when this insect fed on tpMicro-tom plants. Since the injection of the dsRNA resulted in significant gene down-regulation, this result suggests that it is the inaccessibility of the dsRNA, and not the dsRNA sequence used, that prevented the down-regulation of the *v*-*ATPaseA* gene in MMB. Interestingly, a recent study showed that, in whiteflies, which are also sap-sucking insects, the dsRNA expressed in tobacco plastids did not induce gene down-regulation, while the same dsRNA did down-regulate the target gene when expressed in the nucleus [35]. The same study further demonstrated that whiteflies were simply unable to ingest plant plastids, including the P-plastids present in plant phloem. Similarly, a plastid-produced *β-Actin* dsRNA from CPB failed to induce gene down-regulation in green peach aphids, *Myzus persicae*, despite *β-Actin* genes from the two insects having 88.5% sequence identity around the dsRNA sequence [69]. Previously, the nuclear or transient expression of dsRNA in plants was shown to induce gene down-regulation and mortality in sap-sucking insects [70,71,72]. This is most likely due to the transport of siRNA produced in the cytoplasm of plant cells to the plant sap [73,74,75], allowing sap-sucking insects to access siRNA during feeding. Therefore, taken together, our results provide further evidence that plant-mediated RNAi in sap-sucking insects may be limited to the nuclear transformation of plants, as plastid-produced dsRNA cannot be accessed by these insects during feeding.

In summary, we have shown that in vitro-synthesized dsRNA, matching a conserved portion of the cotton mealybug *ATPaseA* gene, can have significant cross-species effects on three insects that use different feeding mechanisms, regardless of the order they belong to. This could have implications for the simultaneous targeting of several insect pests in field applications using highly conserved genes. We also demonstrate that dsRNA is produced in all the plant tissues analyzed in tpMicro-tom, although the concentration in the leaf tissue was the highest, and will probably be most effective at down-regulating a target gene in insects. Finally, we show, for the first time, that when the same dsRNA is presented in Micro-tom plastids, not only a leaf-chewing insect (CPB) but also a lacerate-and-flush feeding insect (BMSB) are affected; however, down-regulation in BMSB was life stage dependent, which may also limit the use of this strategy against this insect. Ultimately, for the application of plastid-produced dsRNA in pest insect control, dsRNAs more closely matching gene targets will be required to achieve debilitating phenotypes, and these should be implemented in species with feeding methods more susceptible to this mode of dsRNA delivery.

## 4. Materials and Methods

### 4.1. Bioassays Using In Vitro-Synthesized dsRNA

The sequence of a 189 bp *v*-*ATPaseA* gene fragment was taken from a previous study [63], and a BLAST analysis revealed that the fragment most closely matches (98%) with the cotton mealybug (*P. solenopsis*; Hemiptera) *v*-*ATPaseA* gene (GenBank: KF384509.1). Synthesized dsRNA representing this *v*-*ATPaseA* gene and a 223 bp *GFP* gene fragment (Supplementary Figure S3D,E) were purchased from RNA Greentech LLC (Frisco, TX, USA) for bioassays.

For CPB, dsRNAs were diluted to 0.5 µg/µL using nuclease-free water, and then potato leaves were dipped in the dsRNA solutions or water for 5–10 s. The leaves were allowed to dry for 2 h, and one treated leaf was placed per Petri dish (lined with moistened Whatman filter paper). Three Petri dishes per treatment group were prepared, and five 1st instar CPB larvae were placed into each Petri dish. Insects were allowed to feed on the leaves ad libitum, and leaves were replaced with fresh treated leaves for three days. After three days, three larvae were pooled per biological replicate and were frozen in liquid nitrogen for RNA extraction and RT-qPCR analysis. A total of three biological replicates (*n* = 3) were conducted per treatment.

For BMSB, dsRNAs were diluted to 1.5 µg/µL, and adult insects were injected with 5 µL of the diluted dsRNA or water using a 10 µL Hamilton glass syringe and 30-gauge needle. Then, insects were placed in 1 L plastic containers and were provided with organic celery and carrot to feed on for three days. At the end of three days, one insect per biological replicate was frozen in liquid nitrogen for RNA extraction and RT-qPCR analysis. Three biological replicates (*n* = 3) per treatment were carried out.

For MMB, dsRNAs were diluted to 6.5 µg/µL. Then, 30 nL dsRNA or water were injected into adult females using glass needles and a Nanoject III Nanoliter Injector (Drummond Scientific, Broomall, PA, USA). The glass needles were pulled from glass capillaries (#3-000-203-G) using a model PP-830 Narishige needle puller (Narishige, Amityville, NY, USA). Injected insects were placed onto WT Micro-tom leaves and allowed to feed for three days. Then, four adults were pooled together to form a biological replicate, and three biological replicates (*n* = 3) were carried out per treatment. Insects were frozen in liquid nitrogen for RNA extraction and RT-qPCR analysis.

### 4.2. Micro-Tom Plant Growth

*Solanum lycopersicum* (var. Micro-tom) seeds were sterilized for 30 min in 25% commercial bleach and rinsed with sterile water 7× before placing them on Murashige and Skoog (MS) medium (4.4 g/L MS Basal Medium with Vitamins, 3% sucrose and 0.7% agar, pH = 5.8) for germination. Leaves from 6–7-week-old plants were used for plastid transformation. Otherwise, 3–4-week-old seedlings were transplanted to general purpose soil and further grown in growth chambers at 24 °C, 16L:8D light:dark cycle, 80% relative humidity, for 4–5 weeks before being used for insect bioassays. When germinating tpMicro-tom seeds, 500 mg/L spectinomycin was added to the medium.

### 4.3. Micro-Tom Plastid Transformation

The plastome transformation vector (pTomCT) and protocol for Micro-tom plastid transformation used in this study have previously been described in detail [37]. Briefly, pTomCT vector was designed to insert an expression cassette into the tomato plastome in a transcriptionally silent intergenic spacer between the *rps12* and *trnV* genes. The expression cassette contains a multi-cloning site (MCS) that is flanked with two inward-facing *rrn* operon promoters (*Prrn*) to drive the transcription of dsRNA, and the *aadA* gene to confer spectinomycin resistance to the transplastomic plants. To clone the dsRNA sequence, a 189 bp *v*-*ATPaseA* gene fragment was obtained by synthesis (Integrated DNA Technologies, Coralville, IA, USA) and used as template for PCR to clone the gene fragment. Primers (Supplementary Table S1) containing *Not* I and *Sal* I restriction sites at their 5′ ends were used to generate PCR products, which were ligated into the MCS on pTomCT. Biolistic particle delivery [51] was used to transform Micro-tom leaves. Bombarded leaves were kept in the dark for 48 h, and then cut into approximately 3 × 3 mm pieces [36] and placed on regeneration medium (RM) (MS medium with 500 mg/L spectinomycin, 1 mg/L 6-benzylaminopurine, 0.1 mg/L 1-naphthaleneacetic acid, 1 mg/L thiamine hydrochloride, and 0.1 g/L myo-inositol). Plates were kept at light intensities of approximately 25 µE [36], and leaf explants were sub-cultured to fresh RM two times until spectinomycin-resistant calli were observed, which took 5–6 months. The calli were cut into small pieces and selected three more times before being placed on shoot-inducing medium (SIM) (MS medium containing 2 mg/L zeatin, 0.1 mg/L IAA, and 500 mg/L spectinomycin). Once the shoots grew 2–3 cm, they were cut and placed on root-inducing medium (MS medium with 500 mg/L spectinomycin) to induce root growth. Finally, the plants were transplanted to soil and grown in greenhouse at 16L:8D light:dark cycle under ambient temperature to collect T_1_ seeds.

### 4.4. DNA and RNA Extraction

Plant tissues were frozen in liquid nitrogen and homogenized using either a TissueLyser (Qiagen, Germantown, MD, USA) or mortar and pestle. Plant DNA extraction was accomplished via CTAB DNA extraction protocol [76]; whereas, plant RNA was extracted using acid phenol:chloroform [77]. Insect RNA was extracted using an RNeasy Mini Kit (Qiagen, Germantown, MD, USA) following the manufacturer’s protocols. To prevent genomic DNA contamination, all RNA samples were treated using an Ambion Turbo RNase-Free DNase kit (Thermo-Fisher, Waltham, MA, USA).

### 4.5. Southern Blot to Confirm Homoplastomy of tpMicro-Tom

The homoplastomy of the tpMicro-tom was confirmed using Southern blotting [51,78]. Briefly, 10 µg Micro-tom DNA was digested using *Mfe* I restriction enzyme to cut the homologous recombination regions in Micro-tom plastome DNA used for transformation and separated on a 0.8% agarose gel. Digested DNA was transferred overnight to an Amersham Hybond membrane (Thermo-Fisher, Waltham, MA, USA). The membrane was then hybridized with a 1181 bp DIG-labelled hybridization probe binding to the plastome recombination region, which was prepared using a PCR DIG Probe Synthesis Kit (Sigma-Aldrich, St. Louis, MO, USA) and primers shown in Supplementary Table S1, using WT Micro-tom DNA as a template. Subsequent blotting steps included washing the membrane twice with 2 × Saline Sodium Citrate (SSC, 20 × SSC is 3.0 M NaCl, 0.3 M sodium citrate, pH = 7.0) buffer + 0.1% SDS at room temperature and three times in 0.5 × SSC + 0.1% SDS at 68 °C. After blocking the membrane with DIG blocking buffer (Roche, Mississauga, ON, Canada), the membrane was incubated with Anti-Digoxigenin-AP Fab antibody solution (Roche, Mississauga, ON, Canada). Finally, the DNA was detected with CSPD chemiluminescent substrate (Roche, Mississauga, ON, Canada).

### 4.6. cDNA Synthesis and End-Point PCR to Confirm dsRNA Production in Micro-Tom

One microgram of total RNA, extracted from leaf, green fruit, red fruit, flower, or root tissue, was denatured at 95 °C for 5 min in the presence of 25 ng random hexamers and then cooled on ice. An Invitrogen Superscript III First-Strand Supermix Kit (Thermo-Fisher, Waltham, MA, USA) was used following the manufacturer’s protocol to synthesize the cDNA. “No reverse transcriptase” (NRT) controls were also included to ensure there was no gDNA carryover. End-point PCR was performed with Taq DNA polymerase and primers specific to *v-ATPAseA* dsRNA (Supplementary Table S1). Additionally, Micro-tom *TIP41* specific primers were used as a positive control for PCR reactions. Finally, PCR products were visualized through agarose gel electrophoresis.

### 4.7. Droplet Digital PCR (ddPCR) to Quantify dsRNA in Micro-Tom Tissues

To compare dsRNA expression in Micro-tom tissues, 100 mg each of leaf, green fruit, red fruit, flower, and root tissues was used for RNA extractions. The tissues were collected from three different tpMicro-tom plants to have three biological replicates (*n* = 3). cDNA was synthesized using 500 ng RNA per sample, as described in the previous section. For ddPCR, primers and probes were optimized following the MIQE guidelines [79,80]. Reactions were performed using ddPCR Supermix for probes (Bio-Rad, Mississauga, ON, Canada), primers and probes at 900 nM and 250 nM, respectively, in a final reaction volume of 20 μL. NRT controls were also run to check samples for gDNA contamination. Droplets were generated with a Bio-Rad QX100 Droplet Generator and PCR was performed using a Bio-Rad T100 Thermocycler with the following temperature profile: initial denaturation at 95 °C for 10 min, 40 cycles of 94 °C for 30 s, 58 °C for 1 min, and 10 min final incubation at 98 °C. Finally, droplets were read on a Bio-Rad QX100 Droplet reader, and data were analyzed using Quantasoft 1.7.4 software. To determine the absolute quantities of dsRNA in each tissue, the Micro-Tom *TIP41* gene was used as a reference gene [42], and the quantities of dsRNA present in 500 ng of RNA from each tissue were calculated by following the guidelines in the droplet digital PCR application guide [81].

### 4.8. Insect Bioassays Using WT and Transplastomic Micro-Tom

CPBs were reared on WT Micro-tom, and eggs were collected for bioassays. Fifty first instar (<24 h old) larvae were individually placed in 100 × 20 mm Petri dishes (lined with moistened Whatman filter paper) and provided with cut WT Micro-tom or tpMicro-tom leaves. Larvae were allowed to feed ad libitum and mortality was recorded for 7 days. In a separate experiment, an additional 50 larvae per treatment were used to determine whether feeding on tpMicro-tom plants has any effects on beetle body weight after 7 days. For RT-qPCR, RNA was extracted from beetles after 7 days of feeding on WT Micro-tom or tpMicro-tom leaves. For RNA extraction, three insects were pooled together to form a biological replicate, and three biological replicates (*n* = 3) were used per treatment.

To prepare Micro-tom leaves for feeding to BMSB adults and 2nd instar larvae, 29 mL plastic solo cups were filled with water and lids were closed. Then, WT Micro-tom or tpMicro-tom leaf petioles were immersed in the water through holes in the lid to keep leaves fresh during bioassays (Supplementary Figure S2). Then, 5 insects were released into 1 L plastics tubs containing the leaves mounted in solo cups. A total of 30 insects per treatment were used, and survivors were collected for RNA extraction and RT-qPCR analysis after 7 days for adults and after 5 days for 2nd instar larvae. For each treatment, one insect was used per biological replicate, and three biological replicates (*n* = 3) were conducted.

For MMB bioassays, Micro-tom leaves were prepared in solo cups as was described for BMSB. Insects were reared on WT Micro-tom, and 10 female adults per biological replicate were collected using a fine art brush and transferred to leaves placed in plastic portion cups. The insects were allowed to feed on the leaves for 7 days ad libitum, after which, 4 insects per biological replicate were collected and frozen in liquid nitrogen for RNA extraction and RT-qPCR analysis. For each treatment, experiments were repeated three times to have three biological replications (*n* = 3). 

### 4.9. cDNA Synthesis from Insect RNA

To synthesize cDNA from insect RNA, an Invitrogen Superscript III First-Strand Supermix Kit (Thermo-Fisher, Waltham, MA, USA) was used, and manufacturer’s protocol was followed for cDNA synthesis. For insects treated with in vitro-synthesized dsRNA or water, 900 ng (CPB) or 800 ng (BMSB and MMB) total RNA was used. For insects that were fed with WT Micro-tom or tpMicro-tom, 1000 ng (CPB and BMSB adults), 350 ng (MMB) or 200 ng (2nd instar BMSB) of total RNA were used.

### 4.10. Quantitative PCR to Measure Gene Down-Regulation in Insects

To analyze mRNA levels in insects, RT-qPCR was performed using a SensiFAST SYBR No-ROX Mix Kit (Meridian Bioscience, Memphis, TN, USA) and a CFX96 Real-Time Detection System (Bio-Rad, Mississauga, ON, Canada) using the following two-step cycling conditions: initial denaturation at 95 °C for 2 min, 40 cycles of 95 °C for 5 s and 60 °C for 30 s, followed by a melt curve analysis. The transcript abundance of the CPB *v*-*ATPaseA* gene was determined using ribosomal protein L8E (*RPL8E*) as a reference gene [82]. For BMSB, *v*-*ATPaseA* mRNA levels were analyzed using 60S ribosomal protein (*60S RP*) gene as reference [65]. In MMB, *v*-*ATPaseA* gene was analyzed using beta-Tubulin (*betaTUB*) as a reference gene [83]. All qPCR primers were tested for amplification efficiencies to comply with the MIQE guidelines [79] and are listed in Supplementary Table S1. Each sample was run in technical triplicate, and relative transcript differences for target genes in the treatment and control groups were estimated using the 2^−ΔΔCt^ method [84]. All raw RT-qPCR data and 2^−ΔΔCt^ calculations are shown in Supplementary Tables S2 and S4.

### 4.11. Comparing Sequence Identities of v-ATPaseA Genes in Insects

To determine the percentage identities of *v*-*ATPaseA* genes in different insects, BLASTn was used to align the 189 bp *v*-*ATPaseA* gene fragment used in this study with the same portion of the *v*-*ATPaseA* genes of the insect species tested. A summary of the alignments is presented in Table 1, and the complete alignments are shown in Supplementary Figure S3. The MMB genome was unavailable at Genbank at the time of manuscript preparation; therefore, the *v*-*ATPaseA* gene sequence for *P. madeirensis* was obtained by sequencing of a 695 bp PCR product obtained using the primers listed in Supplementary Table S1.

### 4.12. Statistical Analysis of the Data

A one-way ANOVA followed by Tukey’s HSD post hoc analysis was performed to determine any statistical differences in *v*-*ATPaseA* mRNA levels in bioassays using in vitro-synthesized dsRNA as well as the dsRNA levels among Micro-tom tissues. For RT-qPCR data, to assess gene down-regulation and insect body weights in WT Micro-tom and tpMicro-tom fed insects, two sample *t*-tests were used. Finally, Kaplan–Meier survival analysis followed by Log-rank tests were used to determine whether differences existed in survival between WT Micro-tom- and tpMicro-tom-fed insects.

## Figures and Tables

**Table 1:** Percentage identities of insect *v*-*ATPaseA* genes to the *v*-*ATPaseA* dsRNA sequence used for Micro-tom transformation.

Insect Species	Order	Accession Number/Availability	% Identity
*P. madeirensis*	Hemiptera	MN364707.1	86
*H. halys*	Hemiptera	XM_014417043	81
*L. decemlineata*	Coleoptera	XM_023156517	76

## Data Availability

GenBank accession numbers are listed for sequence data generated in this study.

## Supplementary Materials

The following supporting information can be downloaded at: https://www.mdpi.com/article/10.3390/ijms23073918/s1.
