# Supplementary material for: Plastid Transformation of Micro-Tom Tomato with a Hemipteran Double-Stranded RNA Results in RNA Interference in Multiple Insect Species
Source: Int J Mol Sci. 2022 Apr 1;23(7):3918. doi: 10.3390/ijms23073918 (PMC8999928; doi:10.3390/ijms23073918)
Supplement: Supplementary file 1 [file ijms-23-03918-s001.zip › ijms-1603720-supplementary.pdf]

Supplementary material

A B C

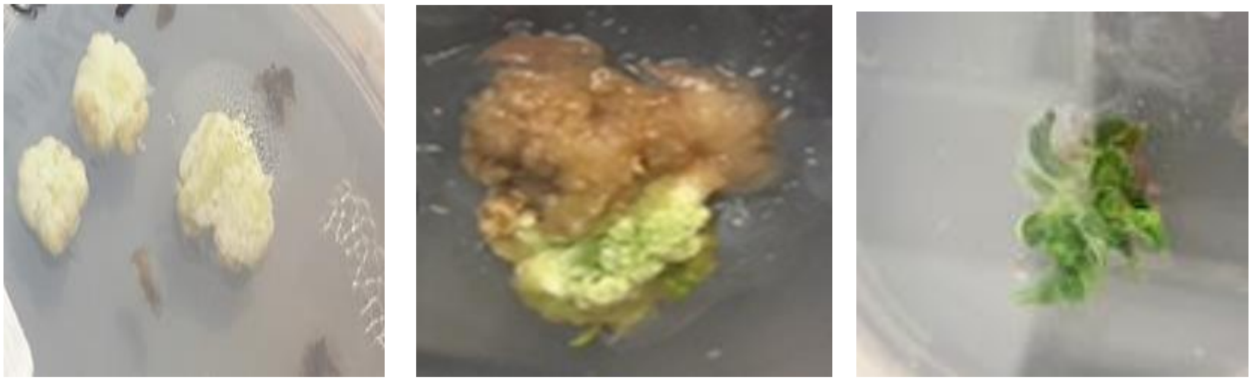

Supplementary Figure S1. Development of transplastomic Micro-tom plants to produce dsRNA for cotton mealybug *v-ATPaseA* gene. (**A**) Transformed Micro-tom leaf explants form pale yellow calli. (**B**) Micro-tom callus after repeated passage on regeneration media. (**C**) Micro-tom callus with shoots after being placed on shoot-inducing medium.

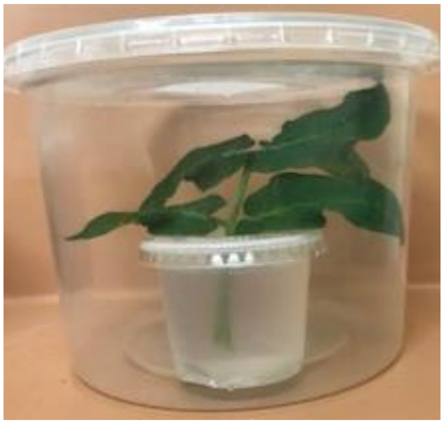

Supplementary Figure S2. An example of leaf petiole preparation for feeding Micro-tom to Madeira mealybugs and BMSB. The leaf petiole was immersed in water (in 29 mL plastic solo cup) and then placed in larger containers for insect feeding.

A

Domain: Data Coding Codon Start: 1	
Halyomorpha halys v-ATPaseA	ATCTGAGAAGTCACCACCAGGAGGTGACACAGCACCGAC
Phenacoccus solenopsis v-ATPaseA	----GAGAAGTCACCACCAGGAGGCGATACCGCGCCAAC
Halyomorpha halys v-ATPaseA	GATGGACACGGATCCTTCTCTCTCTGGGTTACCAAGGCA
Phenacoccus solenopsis v-ATPaseA	AATACTCACAGAACCTTCTCTGTCGGGATTTCCGAGACA
Halyomorpha halys v-ATPaseA	CTTGACTCTGCCAGCACGTTCGTAGAATGAAGCCAATCG
Phenacoccus solenopsis v-ATPaseA	CTTAATTCGACCGGCTCTTTCGTAGAAGGAAGCTAATCT
Halyomorpha halys v-ATPaseA	AGCTCCCAAGTAAGCAGGGTAACCACTGTCGGCAGGCAT
Phenacoccus solenopsis v-ATPaseA	AGCTCCTAAATATGCAGGGTAACCGCTGTCCGCAGGCAT
Halyomorpha halys v-ATPaseA	TTCAGCCAAACGACCGGAAATTTCTCTCAAAGCCTCAGC
Phenacoccus solenopsis v-ATPaseA	CTCTGCCAAACGTCCGGAAATTTCTCTCAG-GCTTCAG-

B

Domain: Data Coding Codon Start: 1	
Leptinotarsa decemlineata v-ATPaseA	CTAAAGTTGCTGAAGTAACGGGATCTGAGAAGTCACCAC
Phenacoccus solenopsis v-ATPaseA	--------------------------GAGAAGTCACCAC
Leptinotarsa decemlineata v-ATPaseA	CGGGTGGAGATACTGCTCCTACGATAGAAACCGATCCTT
Phenacoccus solenopsis v-ATPaseA	CAGGAGGCGATACCGCGCCAACAATACTCACAGAACCTT
Leptinotarsa decemlineata v-ATPaseA	CTCTGTCAGGGTTACCCAAACATTTGACGCGACCAGCAC
Phenacoccus solenopsis v-ATPaseA	CTCTGTCGGGATTTCCGAGACACTTAATTCGACCGGCTC
Leptinotarsa decemlineata v-ATPaseA	GTTCATAGAAAGAGGCAAGACGAGCACCCAAGTAGGCTG
Phenacoccus solenopsis v-ATPaseA	TTTCGTAGAAGGAAGCTAATCTAGCTCCTAAATATGCAG
Leptinotarsa decemlineata v-ATPaseA	GGTAACCAGAATCAGCAGGCATTTCAGCCAAACGTCCTG
Phenacoccus solenopsis v-ATPaseA	GGTAACCGCTGTCCGCAGGCATCTCTGCCAAACGTCCGG
Leptinotarsa decemlineata v-ATPaseA	AAATTTCTCTCAAAGCTTCAGCCCAACGTGATGTAGAGT
Phenacoccus solenopsis v-ATPaseA	AAATTTCTCTC-AGGCTTCAG------------------

C

Domain: Data Coding Codon Start: 1	
Phenacoccus madeirensis v-ATPaseA	GAGGTTACTGGATCTGAGAAATCACCTCCAGGAGGCGAT
Phenacoccus solenopsis v-ATPaseA	---------------GAGAAGTCACCACCAGGAGGCGAT
Phenacoccus madeirensis v-ATPaseA	ACCGCACCGACAATACTTACGGAACCTTCTCGGTCTGGA
Phenacoccus solenopsis v-ATPaseA	ACCGCGCCAACAATACTCACAGAACCTTCTCTGTCGGGA
Phenacoccus madeirensis v-ATPaseA	TTTCCGAGACAATTTGGTTCGACCAGCTCTTTCGTAGAA
Phenacoccus solenopsis v-ATPaseA	TTTCCGAGACA-CTTAATTCGACCGGCTCTTTCGTAGAA
Phenacoccus madeirensis v-ATPaseA	GGAAGCCAATCTGGCTCCTAAATACGCGGGGTACCCGCT
Phenacoccus solenopsis v-ATPaseA	GGAAGCTAATCTAGCTCCTAAATATGCAGGGTAACCGCT
Phenacoccus madeirensis v-ATPaseA	GTCAGCCGGCATCTCGGCTAAACGTCCCGAAATTTCTCG
Phenacoccus solenopsis v-ATPaseA	GTCCGCAGGCATCTCTGCCAAACGTCCGGAAATTTCTCT
Phenacoccus madeirensis v-ATPaseA	AAGAGCTTCGGCCCAACGAGATGTAGAATCAGCCATCAT
Phenacoccus solenopsis v-ATPaseA	CAG-GCTTCAG----------------------------

D

Phenacoccus solenopsis v-ATPaseA	GAGAAGTCACCACCAGGAGGCGATACCGCGCCAACAATA<br>CTCACAGAACCTTCTCTGTCGGGATTTCCGAGACACTTA<br>ATTCGACCGGCTCTTTCGTAGAAGGAAGCTAATCTAGCT<br>CCTAAATATGCAGGGTAACCGCTGTCCGCAGGCATCTCT<br>GCCAAACGTCCGGAAATTTCTCTCAGGCTTCAG

E

GFP dsRNA fragment	A C T T T T C A C T G G A G T T G T C C C A A T T C T T G T T G A A T T A G A<br>T G G T G A T G T T A A T G G G C A C A A A T T T T C T G T C A G T G G A G A<br>G G G T G A A G G T G A T G C A A C A T A C G G A A A A C T T A C C C T T A A<br>A T T T A T T T G C A C T A C T G G A A A A C T A C C T G T T C C A T G G G T<br>A A G T T T A A A C A T A T A T A T A C T A A C T A A C C C T G A T T A T T T<br>A A A T T T T C A G C C A A C A C T T G T C A C T A C T

Supplementary Figure S3. Alignment of 189 bp *Phenacoccus solenopsis v-ATPaseA* gene fragment used in plant transformation with the *v-ATPaseA* genes of insects used in this study. (**A**) Alignment of dsRNA sequence with BMSB *v-ATPaseA* gene; (**B**) Alignment of dsRNA sequence with CPB *v-ATPaseA* gene; **(C)** Alignment of dsRNA sequence with Madeira mealybug *v-ATPaseA* gene; (**D**) 189 bp *Phenacoccus solenopsis v-ATPaseA* gene fragment used in this study; (**E**) 223 bp GFP gene fragment used in *in vitro* bioassays.

Supplementary Table S1. Primers and probes used in this study.

Primer name	5’ to 3’ sequence
**Primers used for cloning of dsRNA sequence into pTomCT vector**	
***Not*I-*v-ATPaseA* F**	GCGGCCGCCTGAAGCCTGAGAGAAAT
***Sal*I-*v-ATPaseA* R**	GTCGACGAGAAGTCACCACCAGG
**Primers used for preparing Dig-labelled probe for Southern blotting**	
**DP*Sl* F**	TCAATCCCTTTGCCCCTCAT
**DL*Sl* R**	TCAACTGCCCCTATCGGAA
**Primers and probes used for confirming dsRNA production and quantification of dsRNA in tpMicro-tom**	
**dsRNA*v-ATPaseA* F**	CGGTTACCCTGCATATTTAG
**dsRNA*v-ATPaseA* R**	CAACAATACTCACAGAACCTTCT
**dsRNA*v-ATPaseA* probe**	[6~FAM]CTTCCTTCTACGAAAGAGCCGGTCGAATT[BHQ1a~Q]
***TIP41* F**	AGACGCCAATGCAACCAAA
***TIP41* R**	AGTGTGGAAGTGCAATACCT
***TIP41* probe**	[6~FAM]TCCTTACAATCCTCCCACTGAATGCAGC[BHQ1a~Q]
**Colorado potato beetle primers used for qPCR**	
***v-ATPaseA* F**	CCAGCTAATCACCCGCTTCT
***v-ATPaseA* R**	CAACCGAAAGCACCGGGAAT
***L8E* F**	GGTAACCATCAACACATTGG
***L8E* R**	TCTTGGCATCCACTTTACC
**Brown marmorated stink bug primers used for qPCR**	
***v-ATPaseA* F**	AACTTCCCCGAGTTTGTTCCA
***v-ATPaseA* R**	TTCAGCGAGGGAGGCTTTA
***60S RP* F**	CCATCAGCAGCTTCTCTTATCA
***60S RP* R**	CTGGCGATGGTGAGGATTT
**Madeira mealybug primers used for qPCR**	
***v-ATPaseA* F**	GCTGAGGTATTACGAGATTTCC
***v-ATPaseA* R**	TTGGCGACGAGAGCTGTTC
***betaTub* F**	CCGACGAACATGGCATTGAC
***betaTub* R**	TGGTTCCGGGTTCAAGATCG
**Primers used for sequencing of Madeira mealybug *v-ATPaseA* gene**	
***v-ATPaseA* F**	CACTATGCTTCAAGTATGGCC
***v-ATPaseA* R**	GACCCCAGAACACTTGCAC

Supplementary Table S2. Data values for Figure 1.

Figure 1: data for CPB fed with in vitro synthesized v-ATPAseA dsRNAfrom cotton mealybug												
	v-ATPase raw Ct			L8e raw Ct			ΔCt			ΔCt average	ΔΔCt	2^-(ΔΔCt)
**treatment**	bio1	bio2	bio3	bio1	bio2	bio3	bio1	bio2	bio3			
H2O	18.59	19.15	19.17	13.93	14.62	14.32	4.66	4.53	4.85	4.68	0	1.000
GFP dsRNA	18.6	18.68	18.88	13.61	13.75	13.69	4.99	4.93	5.19	5.04	0.36	0.781
v-ATPaseA dsRNA	18.98	19.52	19.53	13.7	13.88	13.65	5.28	5.643	5.88	5.60	0.92	0.528
Figure 1: data for BMSB adults injected with in vitro synthesized v-ATPAseA dsRNA from cotton mealybug												
	v-ATPase raw Ct			60S RP raw Ct			ΔCt			ΔCt average	ΔΔCt	2^-(ΔΔCt)
**treatment**	bio1	bio2	bio3	bio1	bio2	bio3	bio1	bio2	bio3			
H2O	17.14	17.16	17.01	16.41	15.56	15.78	0.73	1.6	1.23	1.19	0	1.000
GFP dsRNA	17.49	17.14	17.36	16.57	16.07	16.48	0.92	1.07	0.88	0.96	-0.23	1.173
v-ATPaseA dsRNA	17.54	17.47	17.66	15.8	15.6	15.49	1.74	1.87	2.17	1.93	0.74	0.599
Figure 1: data for MMB injected with in vitro synthesized v-ATPAseA dsRNA from cotton mealybug												
	v-ATPase raw Ct			betaTUB raw Ct			ΔCt			ΔCt average	ΔΔCt	2^-(ΔΔCt)
**treatment**	bio1	bio2	bio3	bio1	bio2	bio3	bio1	bio2	bio3			
H2O	18.43	18.76	18.9	17.4	17.56	17.72	1.03	1.2	1.18	1.14	0.00	1.000
GFP dsRNA	18.59	18.2	18.37	17.56	16.72	17.1	1.03	1.48	1.27	1.26	0.12	0.918
v-ATPaseA dsRNA	19.6	19.08	19.2	16.93	17.05	17.56	2.67	2.03	1.64	2.11	0.98	0.508

Supplementary Table S3. Data values for Figure 3B.

	# of dsRNA molecules			
tissue type	bio1	bio2	bio3	average
tpMicro-tom leaf	74225747	113643556	102254665	9.67E+07
tpMicro-tom flower	12546809	12591925	14862062	1.33E+07
tpMicro-tom green fruit	14847245	14055934	5418000	1.14E+07
tpMicro-tom red fruit	2353042	3818954	253	2.06E+06
tpMicro-tom root	776463	667973	414457	6.20E+05

Supplementary Table S4. Data values for Figure 4A.

Figure 4A: data for MMB adults fed with WT Micro-tom or tpMicro-tom plants												
	v-ATPase raw Ct			betaTUB raw Ct			ΔCt			ΔCt average	ΔΔCt	2^-(ΔΔCt)
**treatment**	bio1	bio2	bio3	bio1	bio2	bio3	bio1	bio2	bio3			
WT Micro-tom	20.79	20.47	20.51	18.93	18.71	18.82	1.86	1.76	1.69	1.77	0	1.000
tpMicro-tom	21.92	21.59	21.17	20.53	19.93	19.75	1.39	1.66	1.42	1.49	-0.28	1.214
Figure 4A: data for BMSB adults fed with WT Micro-tom or tpMicro-tom plants												
	v-ATPase raw Ct			60S RP raw Ct			ΔCt			ΔCt average	ΔΔCt	2^-(ΔΔCt)
**treatment**	bio1	bio2	bio3	bio1	bio2	bio3	bio1	bio2	bio3			
WT Micro-tom	17.1	16.69	16.86	15.55	14.76	15.17	1.55	1.93	1.69	1.72	0	1.000
tpMicro-tom	17.45	16.95	17.33	15.34	15.16	15.15	2.11	1.79	2.18	2.03	0.303333	0.810
Figure 4A: data for BMSB 2nd instars fed with WT Micro-tom or tpMicro-tom plants												
	v-ATPase raw Ct			60S RP raw Ct			ΔCt			ΔCt average	ΔΔCt	2^-(ΔΔCt)
**treatment**	bio1	bio2	bio3	bio1	bio2	bio3	bio1	bio2	bio3			
WT Micro-tom	17.32	18.26	18.25	15.92	17.13	16.65	1.4	1.13	1.6	1.38	0.00	1.000
tpMicro-tom	18.53	19.18	18.97	16.58	17.52	16.85	1.95	1.66	2.12	1.91	0.53	0.691
Figure 4A: data for CPB fed with WT Micro-tom or tpMicro-tom plants												
	v-ATPase raw Ct			L8e raw Ct			ΔCt			ΔCt average	ΔΔCt	2^-(ΔΔCt)
**treatment**	bio1	bio2	bio3	bio1	bio2	bio3	bio1	bio2	bio3			
WT Micro-tom	16.96	17.04	16.96	13.24	13.24	13.17	3.72	3.8	3.79	3.77	0.00	1.000
tpMicro-tom	17.98	18.08	18.28	12.63	12.73	12.81	5.35	5.35	5.47	5.39	1.62	0.325
